# Fatty Acid Composition of Egg Yolk from Chickens Fed a Diet including Marigold (*Tagetes erecta* L.)

**DOI:** 10.1155/2014/564851

**Published:** 2014-12-22

**Authors:** A. Altuntaş, R. Aydin

**Affiliations:** ^1^Department of Animal Sciences, Faculty of Agriculture, Kahramanmaras Sutcu Imam University, Avsar, 46100 Kahramanmaras, Turkey; ^2^Department of Animal Nutrition and Nutritional Diseases, Faculty of Veterinary Medicine, Balikesir University, Campus of Cagis, 10145 Balikesir, Turkey

## Abstract

The objective of this study was to determine the effects of diet supplemented with marigold on egg yolk fatty acid composition and egg quality parameters. Sixty hens were assigned into three groups and fed diets supplemented with 0 (control), 10 g kg^−1^, or 20 g kg^−1^ marigold for 42 days. Eggs collected at the 6th week of the study were analyzed for fatty acid analysis. Laying performance, egg quality parameters, and feed intake were also evaluated. Yolk color scores in the group fed the 20 g kg^−1^ marigold-supplemented diet were found greater than control (10.77 versus 9.77). Inclusion of 20 g kg^−1^ marigold in diet influenced egg weights adversely compared to the control. Diet supplemented with 10 g kg^−1^ or 20 g kg^−1^ marigold increased the levels of C16:0 and C18:0 and decreased levels of C16:1 (n-7) and C18:1 (n-9) in the egg yolk. Also, diet including marigold increased total saturated fatty acids (SFA) and decreased monounsaturated fatty acids (MUFA) in the egg yolk.

## 1. Introduction

The color of egg yolk is an important parameter for consumers and is produced by carotenoid pigments in the feed. The yolk color depends not only on the levels of pigmenting substances, namely, xanthophylls, present in the feed, but also on the type and the ratio of these compounds [1]. There are different sources of xanthophylls used for egg yolk pigmentation. Poultry cannot synthesize these compounds and must obtain carotenoids from their diets [2]. Most of the natural carotenoids that are relevant for poultry pigmentation occur in the free form, but the lutein in* Tagetes *sp. occurs mainly as diesters of palmitic and myristic acids [3]. Since laying hens have the ability to transport 20 to 600 mg g^−1^ of these pigments into their egg yolks from the ingested feed, pigments of either natural or synthetic origins are added to hen diets to achieve the desired egg yolk color [4]. The use of synthetic pigment in the diet increases feed cost. Therefore, natural carotenoids may be considered as pigmenting agents for egg yolk. One of them is marigold which is rich in yellow xanthophylls, mainly lutein and zeaxanthin [5].

Marigold (*Tagetes erecta* L.) is a native plant commonly used in the pigmentation of eggs [6, 7] and contains total carotenoid of 4200 mg/kg [8]. The marigold flour was used at the levels of 16 g kg^−1^, 24 g kg^−1^, or 32 g kg^−1^ for pigmentation in rainbow trouts (*Oncorhynchus mykiss*) and the level of 16 g kg^−1^ of marigold flour was found to be sufficient for pigmentation [9]. There have been a number of studies related to egg yolk pigmentation and egg production in poultry. In a study conducted on laying chickens, marigold flour was added into basal diet at the levels of 8 g kg^−1^ or 12 g kg^−1^ to evaluate the effect of marigold flour on yolk color and egg production in laying chickens [10]. In a recent study, effect of nonsaponified lutein from marigold flour and saponified lutein from marigold flower extract on chicken egg yolk coloration was evaluated [11]. And it was concluded that dietary lutein enhances yolk color at levels of approximately 30 to 40 g kg^−1^ and that saponified lutein from the marigold flour extract appears to be more effective in egg yolk color than nonsaponified lutein from marigold flour [11].

However, there have been no studies reported on the effect of diets supplemented with marigold flour on egg yolk fatty acids in the poultry. Therefore, this study was conducted to evaluate the effects of marigold flour on laying hen performance, yolk fatty acids, and egg properties in chickens.

## 2. Materials and Methods

### 2.1. Birds and Diets

This study was conducted in the research farm at the Kahramanmaras Sutcu Imam University, Turkey. In this study, sixty 80-week-old Hyline-5 laying hens were randomly assigned to three groups with four replicates of five birds each (20 laying hens per group) and fed a diet supplemented with 0 (control), 10, or 20 g kg^−1^ marigold flour (*Tagetes erecta* L.) for 42 days (Table 1). Dried flowers were used after being crushed by using a grinder. Water and feed were provided* ad libitum* during the study. The photoperiod was maintained at 16L:8D throughout the study. Body weights of laying hens were determined at the beginning and end of the study.

Feed consumption was recorded on a subgroup basis at weekly intervals. Feed conversion ratio (FCR) was calculated on weekly basis for every group in the study and was expressed as kilogram of feed consumed per kilogram of egg produced. Eggs were examined for interior and exterior quality. Twelve eggs per group (three eggs per replicate) were collected at the end of the study for measuring egg components and parameters. Egg weight, yolk and albumen index, shape index, albumen weight, yolk weight, and shell weight were measured. Albumen height was measured by a tripod micrometer, albumen length and width were measured by a compass, and then the albumen index was calculated according to the following formula:

(1) Albumen index =albumen height  ×+short diameter of albumen2long diameter of albumen2  wewee+short diameter of albumen2−1×100.

Yolk height and yolk diameter were measured by the same instrument and yolk index was calculated with the following formula: yolk index = yolk height/[(long diameter of yolk + short diameter of yolk)/2] × 100. Shape index was calculated according to the following formula: shape index = (short border/long border) × 100. Shell thickness was measured by a micrometer (Mitutoyo, 0.01 mm, Japan). Shell thickness was a mean value of measurements at three locations on the eggs (air cell, equator, and sharp end). Pigmentation of the egg yolk was measured visually using the Roche color fan (RCF) scale (Roche Ltd., Basel, Switzerland).

### 2.2. Egg Fatty Acid Analysis

Three samples of eggs from each dietary group were obtained randomly for fatty acid analysis at the end of the sixth week of the feeding study. Fatty acid methyl esters (FAMEs) were prepared using methanol/2 N NaOH and extracted with n-hexane and then analyzed by gas chromatography (GC). For this purpose, samples were injected into a Supelcowax 10 column (60 m-0.25 mm i.d., 0.25 lm film thickness; Supelco, Bellefonte, PA, USA) coated with polyethylene glycol. The column was connected to a Hewlett-Packard 5890 Series II (Little Falls, Wilmington, DE, USA) GC equipped with a flame-ionization detector. The oven temperature was programmed as follows: 180°C for 2 min, which increased to 200°C at 2°C/min, held at 200°C for a further 10 min, and then increased to 215°C at 2°C/min and kept there for 10 min. The injector and detector temperatures were 200°C and 250°C, respectively. Helium was used as the carrier gas at a flow rate of 1.5 mL/min. FAME identification was based on retention times compared with thoseof standard FAMEs. C17:0 was used as an internal standard.

### 2.3. Statistical Analysis

One-way analysis of variance (ANOVA) was carried out to determine the effects of marigold flour on egg production, egg weight, feed conversion ratio, egg yolk fatty acid composition, and egg quality parameters in laying chickens. Significance between individual means was identified using the Tukey's multiple range test. Mean differences were considered significant at *P* < 0.05.

## 3. Results

The effect of supplementing diets with marigold flour on body weight, feed consumption, egg production, and egg weight is shown in Table 2. Inclusion of marigold flour in the diets did not affect final body weight, feed consumption, or egg production of the chickens compared to the control group. However, supplementing high level of marigold flour into the diet significantly reduced egg weight compared to the control and the laying hens fed the diet containing the lower level of marigold flour. Inclusion of marigold flour at the high level of marigold significantly decreased egg weight for every week starting from week one compared to the control (not shown here).

The effect of dietary marigold flour on egg parameters in laying hens is shown in Table 3. Inclusion of marigold in the diet had no effect on any egg parameters, except shell strength and yolk color of the eggs. Shell strength of the eggs from hens fed 10 g kg^−1^ marigold flour is significantly greater than the control (*P* < 0.05) and 20 g kg^−1^ marigold supplementation to diet showed a yolk color score of 10.77, which was higher than the control score of 9.77 (*P* < 0.05).

Table 4 shows the fatty acid composition of eggs from laying hens fed diets supplemented with 0, 10, or 20 g kg^−1^ marigold flour. Inclusion of marigold in the diet at either level significantly (*P* < 0.01) increased the levels of C16:0 and C18:0 in the egg yolk while at the same time decreased the levels of C16:1 (n-7) and C18:1 (n-9) (*P* < 0.01). Yolk from hens fed a diet including 20 g kg^−1^ marigold had a higher level of C22:2 (n-6) (*P* < 0.05) when compared to the control. Also, eggs from chickens fed diets including 10 g kg^−1^ or 20 g kg^−1^ marigold flour had higher level of SFA and lower level of MUFA (*P* < 0.05) when compared to the control.

## 4. Discussion

Marigold (*Tagetes erecta* L.) was reported to be a good source of xanthophylls [6, 7] and used for pigmentation of the egg yolks and poultry skin [12, 13]. Recently, it was shown that inclusion of 40 g kg^−1^ of marigold flour in the diet of laying pullets did not reduce external quality of eggs and there were no effects on body weight, hen day egg production, egg weight, or feed conversion rate [14]. The findings of the present study on body weight, hen egg production, and feed consumption are in agreement with those reported in the previous studies [10, 14]. However, in the present study inclusion of 20 g kg^−1^ marigold flour into the diets caused a decrease in egg size compared to the control for an unknown reason.

Inclusion of marigold flour in the diets for pigmentation of egg yolk and poultry meat has been widely documented [1, 6, 7, 10–14]. In the present study, inclusion of marigold in the diet had no effect on egg shell weight, Haugh units, yolk weight, albumen weight, and shell thickness. These findings agree with the previous studies reporting no change in egg quality after inclusion of marigold flour into laying hens' diet [13]. In this study, use of marigold flour in the diet of chicken had no detrimental effect on internal and external quality of egg as well as egg production characteristics.

These results of the present study cannot be compared with data in the literature since there are no reports on the effects of marigold flour on yolk fatty acid composition in poultry. In the present study, inclusion of marigold in the diet significantly increased SFA, mainly C16:0 and C18:0, and decreased MUFA, mainly C16:1 (n-7) and C18:1 (n-9). Similar to the present study, in a study conducted in rainbow trout, it was shown that marigold flour significantly increased C16:0 and C18:0 (*P* < 0.05). In the same study it was shown that inclusion of 18 g kg^−1^ marigold flour in the diet significantly decreased C16:1 (n-7) and C18:1 (n-9) [15]. Therefore, this study may suggest that inclusion of marigold flour in the laying hens' diet increased the ratio of SFA to MUFA by downregulating stearoyl-CoA desaturase, an enzyme converting C16:0 and C18:0 into C16:1 (n-7) and C18:1 (n-9), respectively.

In conclusion, certain fatty acids such as conjugated linoleic acid and conjugated trienoic acids were shown to inhibit SCD [16, 17]. As known, marigold contains naturally occurring conjugated triene fatty acid, calendic acid, to be believed to inhibit the enzyme stearoyl-CoA desaturase. In the future studies, the effects of marigold on stearoyl-CoA desaturase should be investigated.

## Figures and Tables

**Table 1:** Composition of the experimental diets^1^(as dry-matter basis).

Supplementation of *Tagetes erecta* L. (g/kg)			
Feed ingredients	0	10	20
Corn	420.5	420.5	420.5
Barley	120	120	120
Soybean meal	124	124	124
Sunflower meal	100	100	100
Wheat middlings	120	110	100
*Tagetes erecta L*.	0	10	20
Vegetable oil	14	14	14
Limestone	82	82	82
Dicalcium phosphate	12	12	12
Lysine	0.5	0.5	0.5
DL-Methionine	1	1	1
NaCl	2.5	2.5	2.5
Vitamin premix	2.5	2.5	2.5

Total	1,000	1,000	1,000

^1^Diets contained per kilogram of diet: vitamin A, 8000 IU/kg; vitamin D, 1500 IU/kg; riboflavin, 4 mg/kg; cobalamin, 10 ug/kg; vitamin E, 15 mg/kg; vitamin K, 2 mg/kg; choline, 500 mg/kg; niacin, 25 mg/kg; manganese, 60 mg/kg; zinc, 50 mg/kg.

**Table 2:** The effects of *Tagetes erecta* L. supplementation on body weight, feed consumption, egg production, and egg weight.

Supplementation of *Tagetes erecta* L. (g/kg)				
	0	10	20	SEM
Initial body weight (g)	1259	1279	1250	24
Final body weight (g)	1316	1320	1270	28
Feed consumption g/bird	128.1	123.57	117.26	3.87
Egg production (%)	72.0	76.67	71.33	2.93
Egg weight (g)	70.69^a^	70.21^a^	67.97^b^	0.31

^1^Diets were supplemented with 0, 10, or 20 g/kg marigold flower for 6 weeks.

^a,b^Values are expressed as means and without a common superscript are significantly different within a row (*P* < 0.05).

**Table 3:** The effects of diets supplemented with different levels of *Tagetes erecta* L. on the egg parameters^1^.

Supplementation of *Tagetes erecta* L. (g/kg)				
	0	10	20	SEM
Egg weight (g)	69.67	69.99	68.58	1.02
Albumen weight (g/egg)	45.27	44.28	43.06	1.20
Yolk weight (g/egg)	18.80	18.71	18.78	0.23
Shell weight (g/egg)	6.64	6.69	6.72	0.17
Yolk %	27.44	26.98	27.53	0.41
Albumen %	64.49	63.43	62.62	0.87
Shell %	9.58	9.58	9.83	0.24
Albumen index	7.87	7.80	7.32	0.31
Yolk index	41.99	40.86	40.67	0.53
Shape index	73.50	73.41	73.77	0.38
Haugh unit	69.54	68.79	67.85	1.96
Shell thickness (mm*·*10^−2^)	0.36	0.36	0.38	0.01
Shell strength (kg/cm^2^)	1.03^b^	1.32^a^	1.15^ab^	0.09
^ 2^RCF values	9.77^b^	10.40^ab^	10.77^a^	0.18

^1^Eggs were collected in last week of the study and values are expressed as means and without a common superscript are significantly different within a row (*P* < 0.05).

^2^RCF: Roche color fan.

**Table 4:** The effects of *Tagetes erecta* L. supplementation on the fatty acid composition of egg yolks^1^.

Supplementation of *Tagetes erecta* L. (g/kg)				
Fatty acids	0	10	20	SEM
C14:0	0.36	0.35	0.36	0.01
C16:0	24.75^b^	25.76^a^	25.60^a^	0.04
C16:1 (n-7)	3.30^a^	3.15^b^	2.77^c^	0.01
C18:0	7.30^c^	8.02^b^	8.76^a^	0.04
C18:1 (n-9t)	0.13	0.14	0.14	0.01
C18:1 (n-9c)	44.90^a^	43.25^b^	42.87^c^	0.04
C18:2 (n-6)	16.46	16.54	16.59	0.05
*γ*C18:3 (n-6)	0.14	0.15	0.17	0.01
*α*C18:3 (n-3)	0.54	0.50	0.53	0.02
C20:1 (n-9)	0.26	0.26	0.26	0.01
C20:2 (n-6)	0.15	0.15	0.17	0.01
C20:3 (n-3)	0.16	0.16	0.16	0.01
C22:2 (n-6)	1.17^b^	1.19^ab^	1.23^a^	0.01
C22:6 (n-3)	0.38	0.38	0.39	0.01
*∑*SFA	32.41^c^	34.12^b^	34.72^a^	0.03
*∑*MUFA	48.59^a^	46.80^b^	46.04^c^	0.04
*∑*PUFA	19.00	19.08	19.24	0.04
n-6	17.92	18.03	18.16	0.05
n-3	1.08	1.04	1.08	0.01
n-6/n-3	16.59	17.34	16.89	0.25
SFA/MUFA	0.67^b^	0.73^b^	0.75^a^	0.002

^1^Yolk samples of three fresh eggs from each treatment were obtained at the end of the study.

^a,b,c^Means within a row for each variable with no common letter differ (*P* < 0.05).

*∑*SFA = total saturated fatty acids; *∑*MUFA = total monounsaturated fatty acids; *∑*PUFA = total polyunsaturated fatty acids; n-6: omega-6 fatty acids; n-3: omega-3 fatty acids.
